# What is the future role of quantum computation in drug discovery?

**DOI:** 10.1042/ETLS20240012

**Published:** 2025-09-04

**Authors:** Georg Schusteritsch, David W. Wright, Andrew G. Green, Vid Stojevic

**Affiliations:** 1 Kuano Ltd., Hauxton House, Mill Scitech Park, Mill Lane, Cambridge CB22 5HX, United Kingdom; 2 London Centre for Nanotechnology, University College London, London, WC1H 0AH, United Kingdom

**Keywords:** computational biochemistry, drug discovery and design, quantum chemistry, quantum computing

## Abstract

Quantum computing promises to revolutionise a range of scientific fields by providing solutions to previously intractable problems. The link between the technology and applications in chemistry has made drug discovery a natural place to look for ‘low-hanging fruit’. However, until recently, the lack of accessible, scalable hardware has led to comparisons with nuclear fusion. In this article, we describe how developments in both hardware and algorithms are bringing the time closer when quantum computing, as well a the innovations it inspires, adds real value to computational chemistry in real-world applications.

It has been nearly a century since one of the core challenges of understanding quantum mechanics was captured in Dirac’s conundrum: “The fundamental laws necessary for the mathematical treatment . . . [of] the whole of chemistry are thus completely known, and the difficulty lies only in the fact that application of these laws leads to equations that are too complex to be solved” [1]. In more quantitative terms, the cost of simulating quantum mechanical systems can grow exponentially with the size of the system, meaning that we cannot use classical (traditional) hardware to fully understand complex quantum systems. Tackling this obstacle has been a major driver behind efforts to realise scalable quantum computing hardware. This is not, however, the concern of drug hunters who want to understand the more subtle question of to what extent this is true for particular systems of interest. Much of chemistry, for example, can be understood well enough to inform drug design using classical approaches (often just pen and paper) and does not suffer from any exponentially growing costs as we look at increasingly large molecules. Nonetheless, challenges such as property/activity cliffs and modelling compound metabolism highlight areas where more accurate chemical models would be highly valuable [2–4].

The promise of quantum computing is based on the seductive idea of modelling a fundamentally quantum world on a quantum computer. In practice, however, there is a need to create not only this hardware but also algorithms, which link chemistry challenges to achievable computations. Tackling these topics has only come to the forefront of research activities in this millennium, and much of the work lies precisely in the area where classical and quantum meet, offering the potential that algorithm development can accelerate simulations on classical hardware and lead to hybrid approaches, which provide value long before general quantum computers are available. Understanding which systems will benefit from these new approaches and how to map them to relevant hardware relies on understanding:

1. the levels of quantum entanglement – Einstein’s "spooky action at a distance" – present for a particular quantum problem of interest,
2. the nature of the entanglement across the system (e.g. how it varies, which components of the system dominate, etc.), and
3. the reach of classical approaches, and how to make the route to quantum hardware, once it becomes scalable, as efficient as possible.

This implies that developments in this field require cross-disciplinary communication and collaboration. In this article, we describe how this interplay in identifying appropriate challenges and moving towards solutions can affect drug design in the next five years.

Before going into further details, it is important to outline what we mean by a ‘classical’ approach to simulating quantum chemistry. The most commonly used methods are density functional theory (DFT) and the Hartree–Fock (HF) method [5]. DFT is, in principle, an exact theory, but approximations in the form of the so-called exchange-correlation functional have to be introduced. It often strikes an excellent balance between cost and accuracy; however, the approximations introduced in choosing exchange-correlation functionals are not systematically improvable, and it is known to fail entirely for a range of systems [6,7]. HF correctly captures, roughly speaking, the physics behind the traditional bond and atom view of chemistry, but is completely incapable of capturing any complexities linked to the presence of quantum entanglement. It is also a classical approach in the sense that it can be run (very) efficiently on classical computers. Post-Hartree–Fock (post-HF) methods go beyond the approximations inherent to HF with the aim of capturing increasing levels of quantum complexity. This is achieved by systematically improving on the approximations of HF by incorporating electron correlation through more advanced and computationally more expensive techniques such as Møller–Plesset perturbation theory, coupled-cluster approaches and density matrix renormalisation group. While these comprise an area too vast to cover at a meaningful level in a short review, the key takeaway for the present perspective is that many post-HF methods remain efficiently simulable on traditional classical computing resources. As such, they are capable of capturing quantum entanglement for a limited set of real-world systems but, due to Dirac’s conundrum, are bound to fail for systems beyond these limits. At the very extreme end of the post-HF spectrum, one encounters algorithms such as full configuration interaction, which solve the Schrödinger equation exactly, but at an exponential cost on classical computers. The area of maximal interest in terms of next-generation opportunities for drug discovery lies exactly at the frontier between the two, i.e. close to the transition when classical approaches become too expensive to be simulated effectively (begin to incur exponential costs) on classical machines [8–12]. One of the most fruitful approaches to understanding this transition is through the tensor network approach to quantum algorithm design, which efficiently approximates the many-body wavefunctions of chemical systems through networks of tensors and has become the natural language both for deploying algorithms on quantum computers and for enabling quantum simulations to be pushed as far as possible on classical machines. Below, we present three examples where novel algorithm development inspired by the goal of quantum computation is deployed to better understand drug design challenges. We focus only on quantum computing approaches to direct quantum chemistry calculations and specifically exclude methods relying on quantum machine learning. Although advances in the field of quantum machine learning have brought applications in areas such as virtual ligand screening within sight [13], the path to quantum utility is not as clear as for quantum chemistry calculations [,^7^,^14^].

## Extending computational model applicability in chemical space

A key area of interest for quantum computing researchers has been estimating where new methods can outperform classical hardware. For many years, the prototypical benchmark systems in the field of quantum computing were irrelevant to drug discovery, whether too simple (chains of hydrogens) or esoteric (e.g. FeMo cofactor nitrogenase) [15–17]. Recently, Goings et al. [18] considered the more realistic case of cytochrome P450 enzymes (CYPs), a class of heme-containing metalloenzymes with great importance in drug metabolism and steroid biosynthesis. Correctly obtaining relevant properties of iron-containing systems is a significant challenge for standard computational chemistry. For more advanced theoretical approaches on classical and quantum hardware, this involves finding the set of orbitals (the so-called active space) that correctly describes the physics of the system – crucially, the number of orbitals needs to be small for classical approaches, and the question arises at which point, if any, quantum approaches have an advantage in terms of computational cost for the same active space size. Goings et al. [18] use classical algorithms to get an estimate of the sizes of active spaces required to gain useful chemical insights into the system and, at the same time, get an estimate of the classical resources needed. They compared this with quantum resource estimates for quantum phase estimation, a next-generation quantum algorithm that enables energy calculations in quantum chemistry, and they were able to show a crossover at approximately 50 orbitals where quantum computing may become more advantageous. These models correctly captured the key physics of a complicated heme-binding site of around 40 atoms.

## Beyond simple ground state energy calculations

The vast majority of studies of chemistry on quantum computers have thus far concentrated on algorithms to find the electronic ground-state energy of different systems. Computational chemistry in drug discovery, however, often goes beyond simple calculations of the total energy. Cortes et al. [19] consider how to implement symmetry-adapted perturbation theory (SAPT) on quantum hardware. This method allows describing non-covalent interactions in chemistry systems and is thus often employed in better understanding of the binding properties of drugs in enzyme systems. On classical computers, this approach has identified novel interactions not captured without quantum physics [20,21]. Cortes et al. performed quantum resource estimation for a heme and artemisinin complex and discuss existing bottlenecks in their approach. Their work highlights that more complex algorithms going beyond simple total-energy calculations are both feasible and necessary to usefully advance quantum computing for drug discovery.

## Insights into covalent inhibitors via quantum fingerprints

Covalent inhibitors are drugs that irreversibly bind to specific proteins via a covalent bond that forms between the so-called warhead (i.e. the reactive site) of the drug and the proteins. Nearly all the recent successes in covalent inhibitors have been the result of serendipity rather than rational design. The underlying chemistry and particularly the interplay between binding and the chemical reaction that forms the covalent bonds between the warhead and enzyme are not well understood. Thus, computational rational design of novel covalent inhibitors has emerged as a major drug discovery challenge, but computational tools lag behind the rest of small-molecule design. Montgomery et al. [22] addressed the challenge of predicting the properties of covalent warheads within the framework of quantum computing algorithms. In their study, they employ techniques from classical simulations, in particular density matrix embedding theory, and combine these with quantum algorithms to time evolve a given quantum state of the warheads. From the time-evolved states, they extract quantum features used to machine learn the highest occupied molecular orbital and lowest unoccupied molecular orbital levels based on DFT calculations. They highlight the need to identify relevant features, ‘quantum fingerprints’, that can be employed in the analysis of drug discovery systems both as human-interpretable metrics and as input to machine learning approaches. Such quantum fingerprints may aid in gaining better understanding of the differences encountered in the formation of the covalent bonds of covalent inhibitors that the environment of the enzyme may cause, that is, intrinsic reactivities versus pocket-specific reactivities.

## Conclusions

Despite the vast reach of classical methodologies, the key to overcoming many problems within drug discovery is directly related to the difficulty of modelling and understanding the properties of therapeutics and targets at the quantum level. Although a wide range of studies (e.g. using traditional scalable quantum methods such as SAPT and fragment molecular orbital [20,23]) indicating that only quantum-level models can correctly capture the interactions that drive optimal compound binding, the expense of the more sophisticated quantum calculations has limited their application [7]. To achieve wider scale adoption, future approaches based on quantum hardware or shorter term advances in classical algorithms need to either reduce the cost of informing, decision-making or demonstrate a greater advantage over traditional methods in commercially important areas (such as unlocking ‘undruggable’ targets with covalent binders or molecular glues). The ambition to put quantum chemistry on quantum computers is motivating a reimagining of the possibilities and limitations of existing techniques. This drive is bringing solutions to problems previously thought to be beyond the reach of classical hardware, such as predicting drug metabolism in complex metalloenzymes like CYP450, closer to reality. Furthermore, linking these advances to artificial intelligence (AI) offers the potential to develop models that provide decisive information for drug design at a more affordable cost [24].

Taken together, all these advances are creating an impact now. Hardware and algorithmic development are proceeding at pace and transformative changes are being realised on classical hardware sooner than expected, while paving the way towards deployment on scalable quantum hardware of the future. Value is emerging, as near-term methods, hybrid algorithms and quantum-inspired classical techniques have been shown to deliver practical insights and results. This is, in particular, the case for quantum-inspired methods, where the lessons learnt from quantum algorithms have given direct feedback to classical approaches.

The search for quantum advantage has driven a rich and productive interplay between classical tensor network computations and quantum computations, feeding dramatic advances in both. Google’s claims of quantum advantage [25] were overturned by advances in tensor networks [26–28], and IBM’s more recent claims of quantum utility [29] were overturned similarly [30,31]. Delivering on the transformative potential requires collaborative approaches that bring together hardware, software and application specialists from industry and academia. In the U.K, support for this vision is being provided by the National Quantum Computing Centre, which has funded a collaboration between UCL and Kuano, and the covalent binding work by GSK described above.

## Open Access

This article has been published open access under our Subscribe to Open programme, made possible through the support of our subscribing institutions, learn more here: https://portlandpress.com/pages/open_access_options_and_prices#conditional

## Abbreviations

CYPs: cytochrome P450 enzymes
DFT: density functional theory
HF: Hartree–Fock
SAPT: symmetry-adapted perturbation theory
post-HF: post-Hartree–Fock
